# Examining the Correlation between Objective Injury Parameters, Personality Traits, and Adjustment Measures among Burn Victims

**DOI:** 10.3389/fpubh.2015.00049

**Published:** 2015-03-31

**Authors:** Oren Weissman, Noam Domniz, Yoel R. Petashnick, Dalia Gilboa, Tal Raviv, Liran Barzilai, Nimrod Farber, Moti Harats, Eyal Winkler, Josef Haik

**Affiliations:** ^1^The Burn Unit, Department of Plastic and Reconstructive Surgery, Sheba Medical Center (Affiliated to Sackler School of Medicine, Tel-Aviv University), Ramat Gan, Israel; ^2^Bar-Ilan University, Ramat Gan, Israel; ^3^Department of Plastic and Reconstructive Surgery, Assaf Harofeh Medical Center (Affiliated to Sackler School of Medicine, Tel-Aviv University), Tzrifin, Israel; ^4^The Talpiot Medical Leadership Program, Sheba Medical Center, Ramat Gan, Israel

**Keywords:** objective injury parameters, personality traits, burn victims, TBSA, study

## Abstract

**Background:** Burn victims experience immense physical and mental hardship during their process of rehabilitation and regaining functionality. We examined different objective burn-related factors as well as psychological ones, in the form of personality traits that may affect the rehabilitation process and its outcome.

**Objective:** To assess the influence and correlation of specific personality traits and objective injury-related parameters on the adjustment of burn victims post-injury.

**Methods:** Sixty-two male patients admitted to our burn unit due to burn injuries were compared with 36 healthy male individuals by use of questionnaires to assess each group’s psychological adjustment parameters. Multivariate and hierarchical regression analysis was conducted to identify differences between the groups.

**Results:** A significant negative correlation was found between the objective burn injury severity (e.g., total body surface area and burn depth) and the adjustment of burn victims (*p* < 0.05, *p* < 0.001, Table 3). Moreover, patients more severely injured tend to be more neurotic (*p* < 0.001), and less extroverted and agreeable (*p* < 0.01, Table 4).

**Conclusion:** Extroverted burn victims tend to adjust better to their post-injury life while the neurotic patients tend to have difficulties adjusting. This finding may suggest new tools for early identification of maladjustment-prone patients and therefore provide them with better psychological support in a more dedicated manner.

## Introduction

In the United States, more than two million people suffer burn injuries yearly. During 2012, some 25,000 of them required hospitalization. The mortality rate for all burn cases in the US currently stands at 3.7% which places it as the fourth most likely cause of accidental death (1). Moreover, burn injury may deeply affect the psyche as well as the skin. Since the burn victim population imposes a heavy burden on hospitals and healthcare systems, it is important to study the combination of injury components, both physical and psychological, as they have unique significance and consequences. The common burn victim suffers great hardship as a result of the need for painful daily treatments, reconstructive surgeries, intense emotional and psychological load, change in self-image, and a long rehabilitation process (2).

Numerous studies have dealt with the influence of pre-morbid psychopathologies on the rehabilitation process of this patient population. These studies stem from the assumption that the majority of burn victims, compared to the general population, have a background of social and psychological difficulties as many of the burn events occur following suicide attempts or criminal acts (3–7). Other studies have tried to identify different variables that may explain the diversity in quality of rehabilitation outcome and post-injury adjustment among this population (2, 3). The aforementioned studies reflect the notion that there are seemingly two different patient populations recovering from burn injuries. One group is able to adapt efficiently to the new situation following a period of hospitalization and rehabilitation, and can properly function again without any pertinent “emotional scars.” Conversely, some patients have difficulty reintegrating into normal life and need continuous psychological assistance and long-term emotional–functional support (2, 3).

These studies have focused on two types of variables: objective and subjective. The objective variables define the injury objectively (burn size, location, depth, etc.) and parameters relating to demographics and hospitalization (demographic characteristics, length of stay, etc.). Subjective variables include personality variables such as coping ability and the measure of social support. The majority of studies that dealt with the personal implications of the burn injury tried to investigate the various challenges faced by the victims in their rehabilitation process – emotional, behavioral, and social. However, few studies researching these variables and their effects have attempted to trace personality traits that may be beneficial or detrimental to the rehabilitation and adjustment process (8, 9). Research in the field of personality structure is based on the assumption that there are several traits, relatively consistent within time and place, by which it is possible to define a manner of thinking, perception, and behavior (10, 11). The number of fundamental personality traits was suggested initially as 35 (12, 13) and then repeatedly reduced, ultimately to the current model called the “Big Five,” which constitutes one of the contemporary dominant paradigms in the field of personality traits research (14–16). Accordingly, most personality traits may be divided into five categories or five pairs of complementary basic measurements: “N” for “Neuroticism” and maladjustment as opposed to emotional stability (e.g., emotional distress, unrealistic thinking, excessive cravings, inability to defer gratification, and reduced coping capabilities) (17, 18). Factor “E” represents “Extroversion” (as opposed to introversion) and refers to the tendency toward interpersonal interaction, activism, the need for happiness, and the capacity for joy. A person with a high extroversion score is social, active, talkative, and popular (18, 19). “O,” which stands for “Openness,” refers to the search for experiences and the personal value one attributes to oneself. A high score indicates a tendency for curiosity, a developed imagination, willingness to possess unconventional opinions and values, and an ability to experience the full spectrum of feelings (18, 20). “A” is for “Agreeableness,” as opposed to antagonism. Similar to the extroversion factor, this factor also examines the nature of one’s social interactions and where they stand on the axis between compassion and antagonism toward others. A high score is characterized by kindness, good nature, a tendency to trust others, forgiveness, and altruistic actions (20). A low score is usually found among those with narcissistic, paranoid, and anti-social personality disorders, while a high score is found among the avoidant type (18, 21). The last factor is “C” for “Conscientiousness,” which examines the extent of order, diligence, self-control, and motivation in goal-directed behavior and the will to achieve (22). People with a high score tend to be organized, ambitious, diligent in their work, self-motivated, and responsible. On the other hand, the low-scoring people are lazy, irresponsible, indifferent, and motivated by the search for pleasure. Table 1 summarizes the facets of the different traits.

**Table 1:** **Traits and facets of the five-factor model**.

Trait	Facet
Neuroticism	Anxiety
	Angry hostility
	Depression
	Self-conscientiousness
	Impulsiveness
	Vulnerability
Extraversion	Warmth
	Gregariousness
	Assertiveness
	Activity
	Excitement seeking
	Positive emotions
Openness	Fantasy
	Esthetics
	Feelings
	Actions
	Ideas
	Values
Agreeableness	Trust
	Straightforwardness
	Altruism
	Compliance
	Modesty
	Tender mindedness
Conscientiousness	Competence
	Order
	Dutifulness
	Achievement striving
	Self-discipline
	Deliberation

A principal goal of our study is to examine the correlation between personality traits, burn characteristics, and burn victims’ rehabilitation, in order to evaluate the effect of these characteristics on the quality of rehabilitation. Moreover, an early detection of those victims who may have difficulties in the adjustment process can allow caregivers to pay special attention to them and provide additional assistance, thus rendering the rehabilitation process more effective.

## Patients and Methods

Two categories of patients participated in the study: the study group and the control group. The study group was composed of 62 patients hospitalized in the Sheba Medical Center burn unit in Tel Hashomer, Israel, between the years 1996–2001. These patients suffered second and third degree burns from a variety of causes, spanning from 5 to 90% of total body surface area (TBSA). All patients were males hospitalized for more than 2 weeks. Patients were recruited to the study following a period of over a year following their discharge from the hospital. None of the patients had a previously documented mental illness. In order to collect data for the study, questionnaires and informed consent forms were sent to 168 patients who met the aforementioned inclusion criteria. Out of 168, 62 (37%) patients gave consent and filled out the questionnaires. Forty-three of them did so only after repetitive phone calls encouraging them to fill out and return the forms. The control group was composed of 36 men, 23 of whom were reserve army soldiers. In order to examine whether there were significant differences in age and education between the groups, *t*-test analysis was conducted to compare the averages of these variables. For the evaluation of personality traits, the participants completed a questionnaire containing 60 items on the “NEO-Five-Factor Inventory.” The questionnaire was divided into 5 sections, each containing 12 items relative to a distinctive personality trait. A numerical response was sought for each item ranging from 1 (totally true) to 5 (totally false) (23). By calculating the average of each section, five grades are obtained for each personality trait: neuroticism, extroversion, openness, agreeableness, and consciousness. A higher grade means a stronger tendency toward the specific trait. The Cronbach’s alpha score of the five grades ranges between 0.68 and 0.86^1^. The questionnaire model has been repeatedly validated in other studies (10).

As a measure of quality of daily function, the participants filled out an evaluation form drawn from the “West Haven-Yale Multidimensional Pain Inventory” (WHYMPI) questionnaire (24). This test assesses the person’s extent of participation in common activities such as hand washing, shopping, and going out. This questionnaire contains 18 items which participants rank by grading each from 1 (totally agree) to 7 (totally disagree). There are four types of activities: domestic tasks, repairing and renovating, outside activities, and social activities. A global grade was calculated from the means of the four different grades with a Cronbach’s alpha score of 0.79. An additional questionnaire filled by the participants was the “Satisfaction with Life Scale.” It measures general satisfaction according to the participant’s personal judgment without referring to a specific aspect like education or income (25, 26). The form contains five items and, again, the grades for each statement range from 1 to 7. The mean of these five grades creates a single global score. The higher the score, the more satisfied the participant is with his life. The Cronbach’s alpha score of this questionnaire is 0.84. This questionnaire contained questions about age, ethnic origin, socioeconomic status, objective characteristics of burn severity (depth and TBSA), and several details about the subjective aspect of the injury. This questionnaire also includes two subjective measures for the quality of adjustment. One refers to the extent of ability to return to full function post-burn, and the other refers to the extent that the burn is perceived as an obstacle to normal adjustment. These two variables were found to have a very strong correlation (Cronbach’s alpha of 0.85) and were therefore united into a single subjective measure.

The hypothesis of the study was tested by multidimensional methods for variables analysis. These methods included one-way multivariate analysis of variance (Manova) analysis to test the differences between the groups, hierarchical regression coefficients to explain the variance between the different variables, *t*-test for categorical variables, Pearson correlation for the connection between the variables, and Fisher exact *Z*-test for examining the significant differences between the groups.

## Results

The ages of the 62 male patients in our study group ranged from 17 to 82, with an average age of 40.37. The age range in the control group of 36 male participants was 25–51, with an average age of 38.64. Table 2 shows *t*-test analysis comparing the average age and level of education of the two groups. Although the difference in age average between the two groups was not statistically significant, members of the control group were more educated than those of the study group. As seen in Table 3, we found a significant negative correlation between burn severity and the adjustment measures, assessed by the level of satisfaction and daily function. With increasing injury severity, lower satisfaction and daily function levels were found. Data in Table 4 revealed several statistically significant positive and negative correlations. The depth of burn and the extent of trauma, along with unemployment duration and length of stay, correlate positively with neuroticism and negatively with extroversion. Consequently, a patient suffering from a deeper burn, with more extensive trauma, a longer hospitalization, and post-discharge unemployment period, leans toward neuroticism rather than extroversion. Moreover, a negative correlation was found between TBSA, level of trauma and hospitalization duration, and agreeableness. As TBSA, trauma severity, and length of stay decrease, a stronger tendency toward agreeableness is seen.

**Table 2:** **A Comparison of age and education level between the study group and the control group**.

Characteristics	Study group (*n* = 62)		Control group (*n* = 36)		*F* test (1, 94)
	*M*	SD	*M*	SD
Age (years)	40.7	16.16	38.64	12.94	1.66
Education (years)*	4.43	1.56	6.22	1.25	2.92**

*M, mean; SD, standard deviation*.

*Beyond elementary school.

**p value <0.05.

**Table 3:** **Pearson correlation between burn parameters and the adjustment measures amongst burn victims**.

	Satisfaction with life	Daily function
TBSA	−0.22*	−0.40***
Depth	−0.30*	−0.30**

*TBSA, total body surface area*.

*p < 0.05.

**p < 0.01.

***p < 0.001.

**Table 4:** **Correlations between injury parameters and personality traits amongst burn victims**.

	Neuroticism	Extroversion	Openness	Agreeableness	Conscientiousness
TBSA	0.27*	−0.20	−0.18	−0.33**	−0.09
Depth of burn	0.42***	−0.43***	0.16	−0.11	−0.10
Trauma	0.46***	−0.33**	0.01	−0.33**	−0.11
Unemployment period	0.27*	−0.25*	−0.06	−0.21	0.01
Duration of hospitalization	0.54***	−0.34**	−0.14	−0.44***	−0.15

*TBSA, total body surface area*.

*p value <0.05.

**p value <0.01.

***p value <0.001.

One of the study’s goals was to find a link between the different variables and the adjustment capability among burn victims. A hierarchical regression was performed in which the demographic characteristics were entered in the first step, the medical/objective characteristics in the second, and the personality traits in the third. Our tables did not reveal any steps lacking significant contribution for the explanation of variance. As seen in Table 5, several variables are able to explain 22% of the variance of satisfaction and 25% of the daily function among burn victims. The medical/objective variables explain 10% of the satisfaction (specifically, length of stay) and 16% of the daily function (specifically, TBSA). The third step, in which the personality traits were added, has thus contributed 12% for the explanation of the variance of the satisfaction (by neuroticism) and 9% for the explanation of the variance of the daily function (by extroversion). When comparing the relative contribution of the personality traits among the study group and the population of the entire study, the results are virtually the same (8% for the entire population, 9% for the study group). In sum, it seems that neuroticism as a personality characteristic contributes to the variance of satisfaction, whereas extroversion as a characteristic does the same for the daily function.

**Table 5:** **Hierarchical regression coefficients for explanation of the variance of adjustment measures among burn victims**.

Predictors	β	*B*	SEB	*R*^2^
**Satisfaction**
Second step				0.10*
Duration of hospitalization	−0.32*	0.22	0.19	
Third step				0.22**
Duration of hospitalization	−0.10	0.09	0.10	
Neuroticism	−0.41**	0.72	0.25	
**Daily function**
Second step				0.16**
TBSA	−0.40	−0.01	0.01	
Third step				0.25***
TBSA	−0.36**	−0.01	0.01	
Extroversion	0.29*	0.54	0.22	

*TBSA, total body surface area*.

*p < 0.05.

**p < 0.01.

***p < 0.001.

## Discussion

The effect of an injury’s severity on the post-injury adjustment of burn victims should be appreciated and explored. Many studies have investigated that effect as an indicator of rehabilitation prognosis (11, 12, 21, 22, 25). Although intuitively one might expect a strong correlation between the two, some recent studies have found no relationship between the severity of injury and the success of the rehabilitative process (12, 22, 24, 25). Patterson et al. (12) concluded that the reason severity is an important factor in the adjustment process lies in the great value our society gives to esthetics. Gilboa et al. (3) found no connection between the objective components of the injury and the adjustment measures, while conversely, such a connection was found with more subjective measures. In our study, a significant correlation was indeed found between burn severity indexes and the adjustment measures (satisfaction with life and daily function). An increase in the severity of the aforementioned components was correlated with lower levels of adjustment, more severe patient-reported trauma, and longer unemployment periods. On the other hand, these components only account for half of the explained variance of the different adjustment indexes, as seen in Table 5.

The explained variance of satisfaction (22%) was divided between the length of stay and neuroticism. The explained variance of daily function (25%) was shared between TBSA and extroversion. This may lead us to side with a more integrated point of view of the measures, objective and personal, as contributors to the character and the quality of the adjustment. For example, it was postulated that depressive affective disorder following a burn injury is influenced both by the severity of the injury itself and by the style of coping mechanism, such as one’s control of a perceived pain (3). As previously mentioned, a goal of our study was to assess the correlation between the personality traits of the “big five” and adjustment measures. Thus, the greater the correlation between a specific personal trait and the dependent variables, the more the trait is perceived as influential on the adjustment process. In this study, three traits – neuroticism, extroversion, and agreeableness – were found to be the most relevant for our cause. We did not find a significant correlation between openness and the adjustment measures, whereas the trait of conscientiousness correlates significantly with daily function among the study group, according to the Fisher *Z* exact test.

Depending on different life situations, the traits of extroversion and neuroticism also are seen to affect adjustment capability. A higher level of neuroticism consistently correlates with a lesser adjustment capability (satisfaction and daily function). Furthermore, neuroticism accounts for more than half of the variance in the burn victims’ satisfaction measure. On the other hand, a higher level of extroversion is strongly correlated with superior adjustment in these measures. Neuroticism has been associated in many studies with a passive and avoidant way of coping, while extroversion has been linked to active and direct coping (27, 28). These coping styles have even been named “neurotic coping” for neuroticism and “mature coping” for extroversion (29). The role of extroversion as a trait correlated with adaptive coping has been assessed in several studies during the past decade. Described as “a hidden personality factor in coping” (28), it was found to be associated with positive thinking, active problem-centered coping, and optimism (3). Our findings are consistent with this extroversive coping style (“mature coping”) as an essential active style. The influence of extroversion was exceeded only by the influence of TBSA, on the variance of daily function (Table 5). These findings resemble those of Fauerbach et al. (30) who examined the effect of personality traits on the frequency of developing PTSD among the burn victim population. Nevertheless, personality traits are not affected by states of anxiety and fear, as might be perceived, but function as “baseline characteristics” of the patients and are not affected by transient mental states. Therefore, our conclusion is that burn characteristics and patients’ personality traits do predict both the outcome of and the patient’s satisfaction with the rehabilitation process.

Questions about our study’s methodology may be found in two areas: measurement tools and sampling. Adjustment, as mentioned earlier, refers in this study to satisfaction with life and daily function. The latter was measured by the third part of the WHYMPI questionnaire. Unfortunately, measuring adjustment capabilities among the burn victim population has always been problematic (3, 4, 6, 17, 31). Indeed, the WHYMPI questionnaire was not developed specifically for the burn population and might not be fully sensitive to tasks that are difficult for these patients. A unique questionnaire for the burn victim population has recently been developed, but further studies are needed to confirm its validity (3). A second possible methodological criticism relates to participant selection. All the participants were males. Several studies have found significant differences between genders in their quality of coping with burns (4–8, 32, 33) and in coping mechanisms (9). Therefore, we chose to use only one gender in order to exclude a possible bias. Patients with a known history of psychiatric disorders were also excluded to avoid a bias factor regarding personality traits. A few additional disparities between the study and control groups should also be noted – their variant sizes and education levels (see Table 2) and rates of participation (<40% of the potential subjects ultimately participated). Despite these limiting features, we believe their effects on the study’s overall conclusions are minimal. As has also been confirmed elsewhere, the general burn victim population has a higher rate of psychiatric disorders compared to the general population (34).

## Conclusion

This study demonstrates associations among objective injury measures, adjustment capabilities, and personality traits among burn victims. The value of responses to personality questionnaires among burn patients has also been demonstrated. Since some variance could not be explained by the different variables examined here, further studies should assess other potential factors that may influence the rehabilitation and coping process of the burn victim population.

## Conflict of Interest Statement

The authors declare that the research was conducted in the absence of any commercial or financial relationships that could be construed as a potential conflict of interest.

## Footnotes

^1^Montag I (2000). Unpublished data. Tel Aviv University.
