# Corrigendum: Examining the Correlation between Objective Injury Parameters, Personality Traits and Adjustment Measures among Burn Victims

**DOI:** 10.3389/fpubh.2016.00001

**Published:** 2016-02-02

**Authors:** Oren Weissman, Noam Domniz, Yoel R. Petashnick, Dalia Gilboa, Tal Raviv, Liran Barzilai, Nimrod Farber, Moti Harats, Eyal Winkler, Josef Haik

**Affiliations:** ^1^The Burn Unit, Department of Plastic and Reconstructive Surgery, Sheba Medical Center, Ramat Gan, Israel; ^2^Bar-Ilan University, Ramat Gan, Israel; ^3^Psychology Service, Sheba Medical Center (Affiliated to Sackler School of Medicine, Tel-Aviv University), Ramat Gan, Israel; ^4^Department of Plastic and Reconstructive Surgery, Assaf Harofeh Medical Center (Affiliated to Sackler School of Medicine, Tel-Aviv University), Tzrifin, Israel; ^5^The Talpiot Medical Leadership Program, Sheba Medical Center, Ramat Gan, Israel

**Keywords:** objective injury parameters, personality traits, burn victims, TBSA, study

In the original manuscript, there is an error in the author list and Dr. Yoel R. Petashnick was incorrectly given as Dr. Yoel Potachnik in the original publication. This is now corrected to Dr. Yoel R. Petashnick. This error does not change the scientific conclusions of the article in any way. The original files of the article have been updated.

## Conflict of Interest Statement

The authors declare that the research was conducted in the absence of any commercial or financial relationships that could be construed as a potential conflict of interest.

